# COL1A1 Is a Potential Prognostic Biomarker and Correlated with Immune Infiltration in Mesothelioma

**DOI:** 10.1155/2021/5320941

**Published:** 2021-01-04

**Authors:** Cangang Zhang, Shanshan Liu, Xin Wang, Haiyan Liu, Xiaobo Zhou, Haibo Liu

**Affiliations:** ^1^Department of Pathogenic Microbiology and Immunology, School of Basic Medical Sciences, Xi'an Jiaotong University, Xi'an, 710061 Shaanxi, China; ^2^Health Science Center, Xi'an Jiaotong University, Xi'an, 710061 Shaanxi, China; ^3^Department of Hematology, The First Affiliated Hospital of Xi'an Jiaotong University, Xi'an, 710061 Shaanxi, China

## Abstract

**Objective:**

Mesothelioma (MESO) is a rare tumor derived from mesothelium cells. The aim of this study was to explore key candidate genes and potential molecular mechanisms for mesothelioma through bioinformatics analysis.

**Methods:**

The MESO expression profiles came from the Gene Expression Omnibus (GEO) and The Cancer Genome Atlas (TCGA) databases. The differences in the infiltration levels of immune cells between MESO and normal tissues were assessed using CIBERSORT. Differentially expressed genes (DEGs) were identified by comprehensive analysis of multiple datasets. A protein-protein interaction (PPI) network was constructed, and a hub gene COL1A1 was selected for MESO. The expression and mutation of COL1A1 in MESO were analyzed in the cBioPortal database. The correlation between COL1A1 expression and immune cell infiltration was evaluated using the TIMER database. Gene Set Enrichment Analysis (GSEA) of COL1A1 was then performed. Finally, Kaplan-Meier survival analysis was presented to predict the survival times between high and low COL1A1 expression groups for MESO patients.

**Results:**

There were distinct differences in the infiltration levels of immune cells between MESO and normal tissues. A total of 118 DEGs were identified by comprehensively analyzing three expression profile datasets. COL1A1, a hub gene, was identified to be highly expressed in MESO compared to normal tissues. COL1A1 genetic mutation occurred in 9% of MESO samples, and amplification was the most common type of mutation. COL1A1 expression was significantly correlated to the infiltration levels of CD4+ T cells, macrophages, and neutrophils. GSEA results indicated that COL1A1 could be involved in key biological processes and pathways like extracellular matrix and PI3K-Akt pathway. Patients with high COL1A1 expression usually experienced shorten overall survival time than those with its low expression.

**Conclusion:**

Our findings revealed that COL1A1 could become a potential prognostic biomarker for MESO, which was significantly related to immune cell infiltration.

## 1. Introduction

MESO is a rare tumor that mainly originates from mesothelial cells [1]. The incidence of MESO is on the rise in recent years due to asbestos exposure [2]. Malignant pleural mesothelioma (MPM) exhibits the highest incidence (81%) and the worst prognosis among all cases of MESO [1]. MESO can also occur in membranous structures in other parts, including peritoneum (9%), pericardium, and testicular sheath [3, 4]. The histological subtypes of MESO are comprise of epithelial (the most common), sarcomatoid, and biphasic (mixture of epithelial and sarcomatoid) [5, 6]. Patients with epithelial type often experience better prognosis than those with mixed or sarcomatous type [5, 6]. Most patients with malignant MESO are at an advanced stage at the time of diagnosis, with a median overall survival of only 1 year and the 5-year overall survival rate of about 10% [7–9].

At present, surgical resection and subsequent chemotherapy are the main treatment strategies for MESO patients. Systemic chemotherapy based on cisplatin and pemetrexed is the main palliative treatment for most malignant pleural MESO patients [10]. MPM patients' prognosis is usually poor, with a median survival of 12 months for patients receiving pemetrexed and cisplatin chemotherapy [11]. Immunotherapy has achieved significant results in a variety of tumors [12], which is expected to be used to improve the prognosis of MESO. However, whether the interaction between MESO and the immune system could affect the prognosis of MESO patients remains unclear. Under normal physiological conditions, the immune system strictly controls cell proliferation and apoptosis with the help of immune cells [13]. In addition, tumor-infiltrating lymphocytes (TILs) play an important role in tumor-related immune responses [14]. Several studies have shown that TILs can effectively improve the prognosis of various tumors [15–18]. Therefore, it is of great clinical significance to explore the infiltration of immune cells in MESO.

The purpose of this study was to identify key gene related to MESO patients' clinical outcomes and to explore the correlation between its expression and immune cell infiltration, which could help us determine the treatment decisions and predict the prognosis for patients. A hub gene, COL1A1 was identified to be upregulated in MESO tissues compared to normal tissues and significantly related to the infiltration levels of various immune cells. For MESO patients, high COL1A1 expression indicated a poorer prognosis than its low expression. Hence, it could become a potential prognostic biomarker for MESO.

## 2. Materials and Methods

### 2.1. Microarray Data

**Figure 1:**
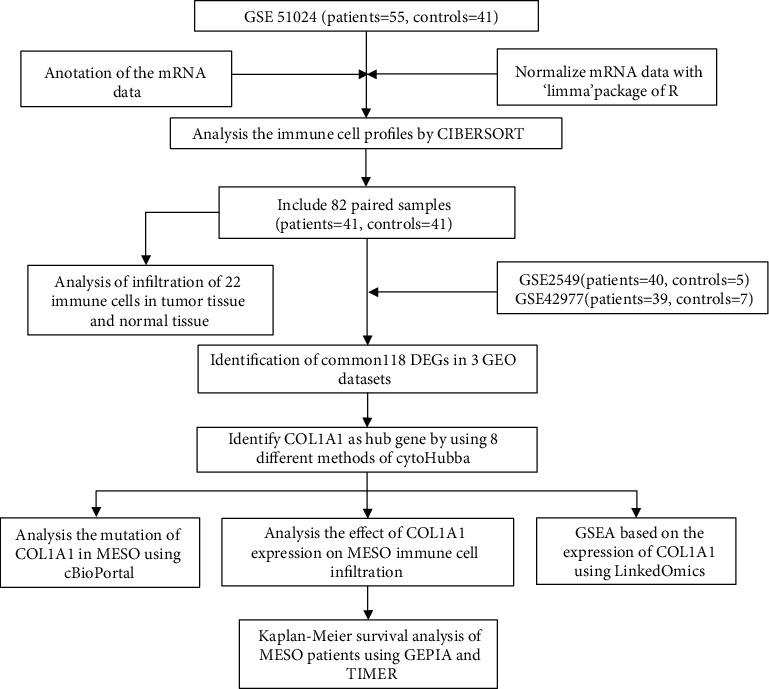
The flowchart in this study.

The GEO (https://www.ncbi.nlm.nih.gov/geo) is a public functional genomics data repository of high-throughput gene expression data, chips, and microarrays [19]. Three gene expression datasets including GSE51024 (Affymetrix Human Genome U133 Plus 2.0 Array) [20], GSE42977 (Illumina HumanRef-6 v2.0 expression bead chip) [21], and GSE2549 (Affymetrix Human Genome U133A Array) [22] were downloaded from the GEO database. The GSE51024 dataset contained 55 MESO samples and 41 normal samples. The GSE42977 included 39 MESO samples and 7 normal samples, and the GSE2549 included 40 MESO samples and 5 normal samples. Normal tissues were obtained from the adjacent cancer of the same MESO patient during surgery. The probes were transformed into the corresponding gene symbol according to the annotation information.  Figure 1 depicted the workflow of this study. The clinical information of MESO patients in the GSE51024 dataset was listed in Supplementary table 1.

### 2.2. Identification of DEGs

The DEGs between MESO and normal samples were screened using the GEO2R (https://www.ncbi.nlm.nih.gov/geo/geo2r). GEO2R is an interactive web tool that enables users to compare two or more datasets in a GEO sequence through experimental conditions. Benjamini and Hochberg's false discovery rates were applied to adjust *p* values [23]. The screening criteria of DEGs were as follows: ∣log fold change (FC) | >1 and adjusted *p* value < 0.05. The common DEGs were overlapped among the three datasets (GSE51024, GSE42977, and GSE2549).

### 2.3. Cell Type Identification by Estimating Relative Subsets of RNA Transcripts (CIBERSORT)

CIBERSORT can accurately quantify the percentage of various tumor-infiltrating immune cells (TIICs) under the complex “gene signature matrix” based on 547 genes [24]. In the current study, the immune infiltration of 22 kinds of immune cells for each sample was assessed by the LM22 signature file, with the preset signature matrix at 100 permutations. The distribution of 22 subtypes of TIICs was then presented, followed by calculation of correlation coefficient, *p* value, and root mean squared error (RMSE). The *p* value < 0.05 represents a statistical connotation of deconvolution outcomes for all cell subsets for each sample and has been useful for less precise exclusion of outcomes. Finally, 41 MESO samples and 41 paired control samples which met the required *p* value < 0.05 were selected for further analysis.

### 2.4. Protein-Protein Interaction PPI Networks and Hub Genes

A PPI network was constructed based on DEGs using the STRING (version 11.0; http://string-db.org) database. STRING is an online database used to predict interactions between proteins [25], which is essential for recognizing the mechanisms of cell activities at the molecular levels in cancer progression. The cut-off value was defined as an interaction score (median confidence) of 0.4. The PPI network was visualized by the Cytoscape software (version 3.7.2; http://www.cytoscape.org/). Hub genes were ranked by the cytoHubba plug-in [26].

### 2.5. Immune Cell Infiltration

The mutations of COL1A1 across 87 MESO samples were assessed in the cBioPortal database (https://www.cbioportal.org/) [27]. In addition, the correlation between COL1A1 expression and the abundance of 6 types of infiltrating immune cells (B cells, CD4+ T cells, CD8+ T cells, neutrophils, macrophages, and dendritic cells) was calculated among MESO samples via The Tumor Immune Estimation Resource (TIMER) algorithm database (https://cistrome.shinyapps.io/timer/) [28]. TIMER is a powerful online tool that can analyze the infiltration of immune cells in different tumors [28].

### 2.6. Gene Set Enrichment Analysis (GSEA)

The LinkedOmics database (https://www.linkedomics.org/) contains multiomics and clinical data across 32 cancer types and 11,158 patients from The Cancer Genome Atlas (TCGA) project [29]. Furthermore, the database has numerous collated data available for download and has a very powerful online analysis function. In this study, GSEA was presented to study the differences in the high and low expression of COL1A1 groups for MESO based on this powerful database, including Gene Ontology (GO) and Kyoto Encyclopedia of Genes and Genomes (KEGG).

### 2.7. Survival Analysis

MESO patients were divided into the high- and low-expression groups based on the median value of the COL1A1 expression. The differences in overall survival and recurrence-free survival were analyzed between the two groups and Kaplan–Meier curves were depicted via the online tool Gene Expression Profiling Interactive Analysis (GEPIA) [30]. The impact of 6 types of immune cell infiltration on the overall survival of MESO patients was also analyzed using the TIMER.

### 2.8. Statistical Analysis

All statistical analysis was carried out using R language (version 3.6.2) packages including “limma,” “CIBERSORT,” “pheatmap,” “corrplot,” “vioplot,” and “ggplot2.” *p* value < 0.05 was considered statistically significant.

## 3. Results

### 3.1. The Distribution of TIICs in MESO and Matched Normal Tissues

**Figure 2:**
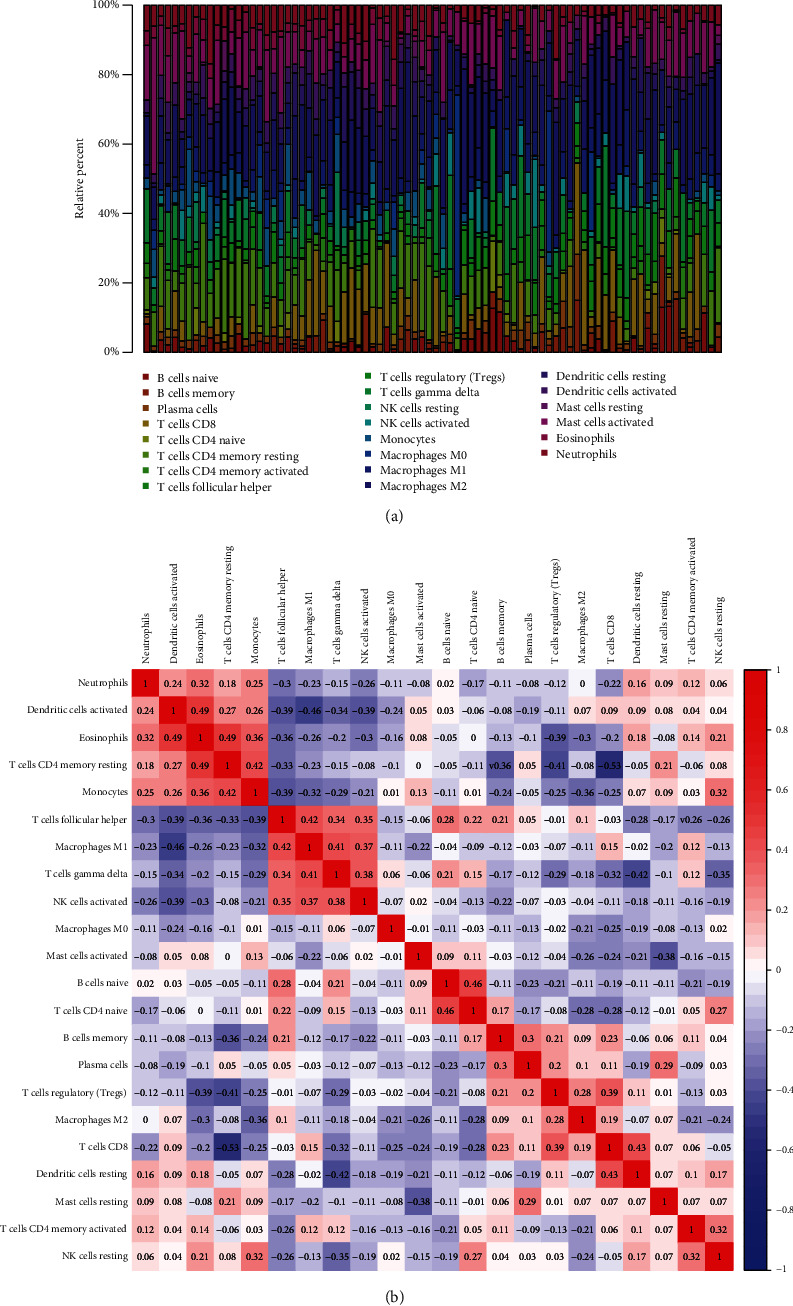
Landscape of immune cell infiltration in MESO and normal tissues by CIBERSORT. (a) The composition of 22 kinds of immune cells in MESO and normal tissues. (b) Heat map visualizing the correlation between 22 kinds of immune cells across MESO and normal samples.

**Figure 3:**
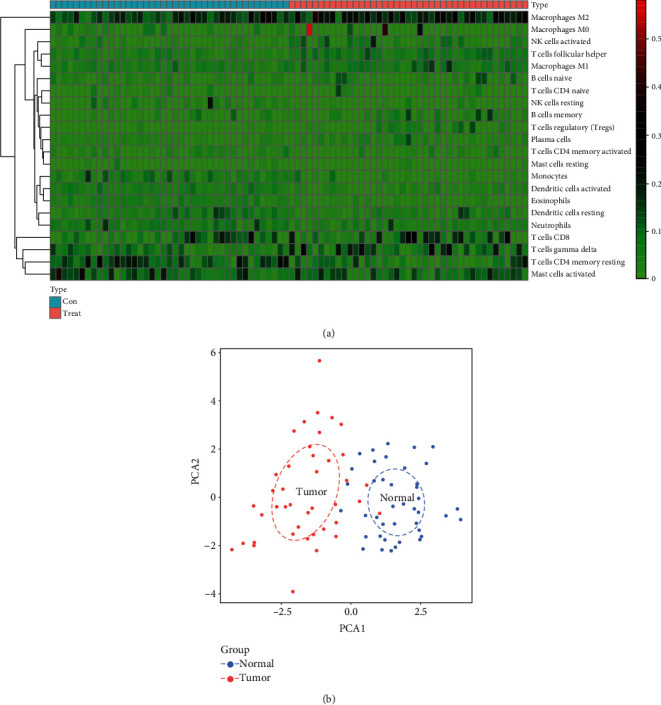
The differences in immune cell infiltration between MESO and normal tissues. (a) Heatmap showing the difference in infiltration levels of 22 kinds of immune cells between MESO and normal samples. (b) Principal component analysis depicting the distinction between MESO and normal tissues based on these immune cells. The blue dots indicate normal samples, and the red dots indicate tumor samples.

We investigated the differences in 22 subpopulations of TIICs between MESO tissues and normal tissues using the CIBERSORT algorithm. Finally, 41 MESO tissues and the paired control tissues met the screening criteria (*p* < 0.05).  Figure 2(a) illustrated the distribution of 22 kinds of TIICs in 41 MESO tissues and control tissues. As shown in Figure 2(b), the fractions of different immune cells were weakly to moderately correlated in MESO tissues. Among them, there was the strongest positive correlation in infiltration mode between eosinophils and activated dendritic cells (coef = 0.49), alongside with correlation between CD4 memory resting T cells and eosinophils (coef = 0.49). Furthermore, there was the strongest negative correlation in infiltration mode between CD8+ T cells and CD4 memory resting T cells (coef = −0.53). On the whole, some immune cells were relatively abundant in tumor tissues, and some were relatively abundant in normal tissues (Figure 3(a)). For example, the infiltration levels of monocytes, activated dendritic cells, and eosinophils in normal tissues were obviously higher than those in MESO tissues. We further probed into whether the two tissue types could be differentiated by these 22 immune cells. Based on the differences in infiltration of 22 types of immune cells, tumor tissues were clearly distinguished from normal tissues by principal component analysis (Figure 3(b)).

### 3.2. Infiltration Levels of Immune Cells Are Distinct in Normal and MESO Tissues

**Figure 4:**
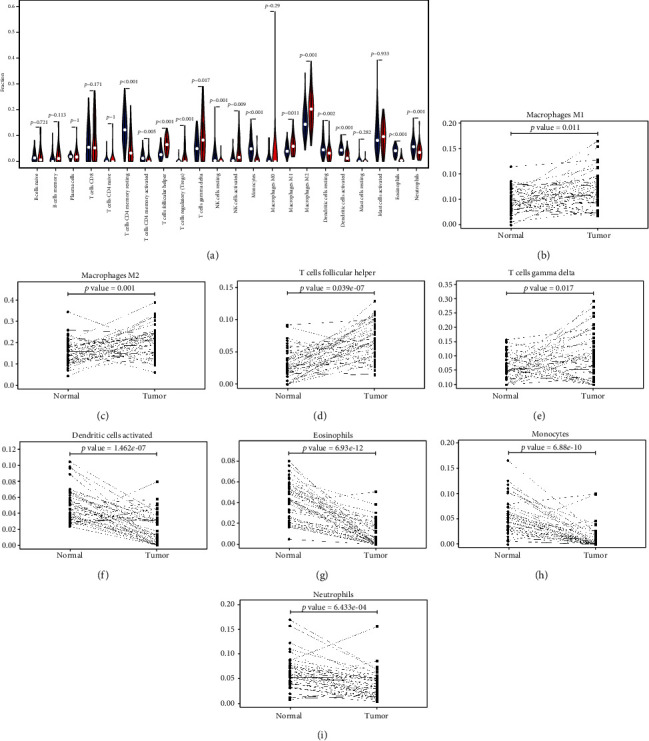
The differences in immune cell infiltration between MESO and paired normal tissues. (a) Violin plots visualizing the distributions of TIICs between MESO and paired normal tissues. Blue represents normal samples and red indicates MESO samples. The infiltration levels of (b) M1 macrophages, (c) M2 macrophages, (d) T cell follicular helper, (e) T cell gamma delta, (f) activated dendritic cells, (g) eosinophils, (h) monocytes, and (i) neutrophils were different between the two groups.

We further determined the difference in immune cell infiltration between MESO and normal tissues. The average proportion of each immune cell type in MESO tissues and paired normal tissues was evaluated, as shown in Figure 4(a). Our data revealed that the proportions of Treg cells (*p* < 0.001; Figure 4(a)), M1 macrophages (*p* = 0.011; Figure 4(b)), M2 macrophages (*p* = 0.001; Figure 4(c)), T cell follicular helper (*p* < 0.001; Figure 4(d)), and T cell gamma delta (*p* = 0.017; Figure 4(e)) were all distinctly higher in MESO tissues compared to normal tissues. Additionally, we observed that the infiltration levels of resting CD4+ memory resting T cells (*p* < 0.001; Figure 4(a)), CD4+ memory activated T cells (*p* = 0.005; Figure 4(a)), resting dendritic cells (*p* = 0.002; Figure 4(a)), activated dendritic cells (*p* < 0.001; Figure 4(f)), eosinophils (*p* < 0.001; Figure 4(g)), monocytes (*p* < 0.001; Figure 4(h)), and neutrophils (*p* < 0.001; Figure 4(i)) in normal tissues were all significantly higher than MESO tissues.

### 3.3. Identification of DEGs and Hub Genes for MESO

**Figure 5:**
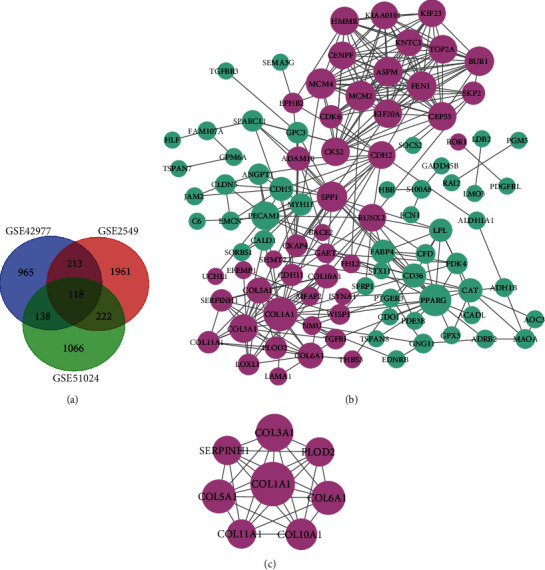
Identification of DEGs and hub genes for MESO. (a) Venn diagram depicting common DEGs among 3 different GEO datasets with the threshold of ∣logFC > 1∣ and adjusted *p* value < 0.05. (b) A PPI network based on DEGs using Cytoscape. (c) A key module based on the PPI network by the MCODE plugin. Light purple suggests upregulated genes, and light green suggests downregulated genes.

GEO2R was used to identify DEGs between MESO and normal tissues. With the threshold of ∣logFC | >1 and adjusted *p* value < 0.05, DEGs (1,983 in GSE51024, 1,470 in GSE42977, and 2,851 in GSE2549) were identified for MESO tissues compared to normal tissues. The overlap among the three datasets contained 118 DEGs, as shown in the Venn diagram (Figure 5(a)), composed of 66 downregulated genes and 52 upregulated genes in MESO tissues compared to normal tissues. Then, these DEGs were analyzed in the STRING database. A PPI network was constructed by the Cytoscape software, including 97 nodes and 299 edges (Figure 5(b)). The degree of each node was calculated. Among them, COL1A1 had the highest degree according to the eight ranked methods using cytoHubba (Table 1). The plugin MCODE of the Cytoscape was used to establish the key module based on the PPI network (Figure 5(c)). In this module, COL1A1 was at the core position and was most connected to other genes.

### 3.4. Expression and Mutation of COL1A1 in MESO

**Figure 6:**
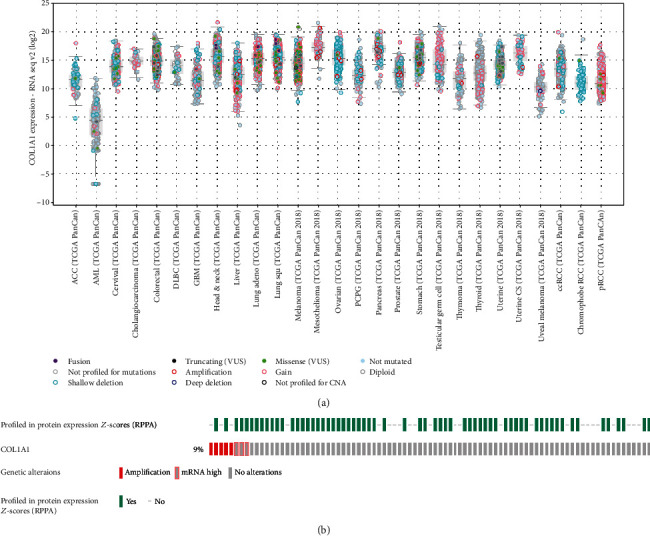
The COL1A1 expression and mutation in MESO tissues from the cbioportal database. (a) The expression of COL1A1 across 27 kinds of cancers. (b) COL1A1 genetic alteration in MESO using reverse-phase protein arrays (RPPA).

In the cBioPortal database, we analyzed the expression of COL1A1 across 27 kinds of cancers. As shown in Figure 6(a), COL1A1 exhibited a higher expression in MESO than normal tissues (other tumors). Subsequently, 87 MESO samples (TCGA, Firehose Legacy) were selected to analyze the COL1A1 genetic alteration. It was found that the COL1A1 genetic alteration occurred 9% across 87 MESO patients (Figure 6(b)). Among them, amplification was the most common type of mutation. Furthermore, using the reverse-phase protein arrays (RPPA), we found that COL1A1 protein was frequently expressed in MESO tissues.

### 3.5. COL1A1 Expression Is Associated with Immune Cell Infiltration in MESO

**Figure 7:**
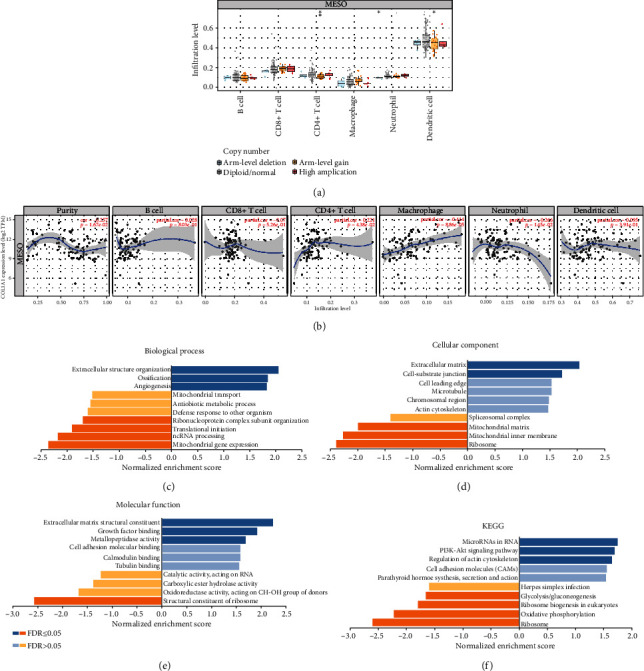
COL1A1 expression is associated with immune cell infiltration in MESO. (a) The correlation between somatic copy number alterations and immune cell infiltration. (b) The correlation between COL1A1 expression and immune cell infiltration in MESO samples. Functional enrichment analysis of COL1A1 including (c) GO biological process, (d) GO cellular components, (e) GO molecular function, and (f) KEGG.

Using the TIMER, we analyzed the correlation between different somatic copy number alterations and immune cell infiltration in MESO samples. As shown in Figure 7(a), our data indicated that somatic copy number alterations were significantly correlated to the infiltration of CD4+ T cells (*p* < 0.01), neutrophils (*p* < 0.05), and dendritic cells (*p* < 0.05). Samples with diploid/normal exhibited the highest infiltration levels of CD4+ T cells and dendritic cells. The correlation between COL1A1 expression and immune cell infiltration was analyzed across patients with MESO (Figure 7(b)). COL1A1 expression was distinctly correlated to tumor purity (cor = −0.257; *p* = 1.67*e* − 02), CD4+ T cells (cor = −0.221; *p* = 4.38*e* − 02), macrophages (cor = 0.414; *p* = 8.86*e* − 05), and neutrophils (cor = −0.266; *p* = 1.45*e* − 02). GSEA including GO and KEGG was performed for COL1A1. Our results showed that COL1A1 was significantly related with key biological processes such as extracellular structure organization, ossification, and angiogenesis (Figure 7(c)). COL1A1 was correlated with several critical cellular components like extracellular matrix and cell-substrate junction (Figure 7(d)). COL1A1 could be involved in regulating molecular functions of extracellular matrix structural constituent, growth factor binding, and metallopeptidase activity (Figure 7(e)). As for KEGG pathways, COL1A1 was associated with microRNAs in cancer, PI3K-Akt signaling pathway, and regulation of actin cytoskeleton (Figure 7(f)).

### 3.6. COL1A1 Could Be a Potential Prognostic Marker for MESO Patients

**Figure 8:**
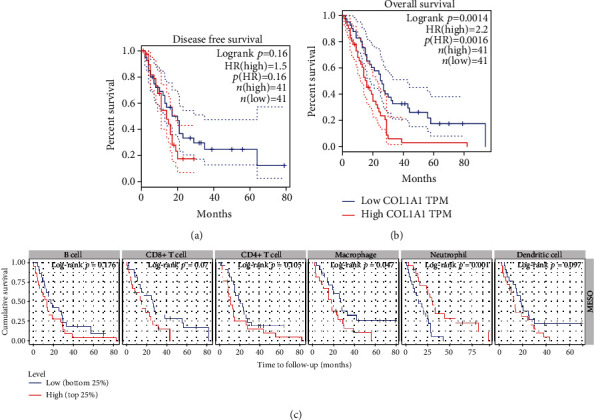
COL1A1 expression is associated with MESO patients' prognosis. (a) Disease-free survival and (b) overall survival analyses of COL1A1 expression for MESO patients using the GEPIA online platform. (c) The correlation between the infiltrations of 6 immune cells and MESO patients' prognosis.

Cox proportional hazard model was constructed to assess the prognostic value of COL1A1 expression in the survival of MESO patients using the TIMER, as listed in Table 2. Among other clinicopathological factors, COL1A1 had a significant association with prognosis of MESO patients (p<0.0001). To further explore the association between COL1A1 expression and patients' survival, MESO patients were divided into the high- and low-expression groups based on the median value of COL1A1 expression using the online tool GEPIA. The data showed that there was no significant difference in disease-free survival between the high- and low-expression groups of COL1A1 for MESO patients (Figure 8(a)). However, patients with high COL1A1 expression indicated poorer overall survival time compared to those with its low expression (*p* = 0.0014; Figure 8(b)). We also evaluated the correlation between immune cell infiltration and MESO patients' prognosis. As shown in Figure 8(c), high levels of neutrophil infiltration (*p* = 0.001) and low levels of macrophage infiltration (*p* = 0.047) distinctly predicted better clinical outcomes.

## 4. Discussion

Based on the MESO dataset from the GEO database, we found the differences in immune cell infiltration between MESO tissues and matched normal pleural tissues. By combining two other GEO datasets, we identified 118 DEGs for MESO. A hub gene, COL1A1, was identified based on the PPI network. Through the cBioPortal database, COL1A1 had a high genetic alteration (9%) across MESO samples, and amplification was the most common type of mutation. COL1A1 expression was markedly associated with MESO patients' clinical outcomes, which could become a potential prognostic marker.

High expression of COL1A1 has been shown to be closely linked to the progression of various cancers [31–34]. Consistently, in this study, the expression of COL1A1 in tumor tissues was distinctly higher compared with normal tissues. Furthermore, the results showed that the mutation of COL1A1 had a distinct correlation with the infiltration of neutrophils, CD4+ T cells, and dendritic cells. Meanwhile, COL1A1 expression exhibited a significant association with tumor purity, CD4 T cells, macrophages, and neutrophils. Thus, COL1A1 expression might be related to tumor immune microenvironment.

In GSEA results, we found that extracellular matrix (ECM) and cell-substrate junction were significantly enriched in the high COL1A1 expression group, indicating that COL1A1 might affect the local infiltration of immune cells by affecting ECM, which was consistent with the results of Homan et al. [35]. For KEGG pathway enrichment analysis results, microRNAs in cancer, PI3K-Akt signaling pathway, and regulation of actin cytoskeleton were related to high COL1A1 expression. A previous study has shown that the PI3K-Akt pathway is frequently activated in MPM [36]. These results indicated that high expression of COL1A1 could promote the progression of MESO. Cox proportional hazard model analysis results demonstrated that COL1A1 expression was in relationship with MESO patients' prognosis. High COL1A1 expression usually predicted a poorer prognosis for MESO patients. Thus, COL1A1 expression could be a promising immune-related prognostic marker for MESO.

Due to the neutrophils' short life span, it is difficult to isolate and study neutrophils associated with tumors, especially human tumor-associated neutrophils (TANs). Up to now, it remains unclear whether neutrophils could promote tumor growth [37, 38] or not [39–41]. In this study, we found that for patients with MESO, high levels of neutrophil infiltration predicted longer overall survival time. Moreover, the expression of COL1A1 was negatively correlated with neutrophil infiltration. Hence, it is of importance to further explore the association between neutrophil infiltration and MESO patients' prognosis. Consistent with previous studies, we found that high levels of macrophage infiltration predicted a worse clinical outcome for MESO patients [42–44]. Moreover, patients with high expression of COL1A1 indicated a higher level of macrophage infiltration. Taken together, these findings indicated that COL1A1 might affect the survival of patients partly by mediating the local infiltration of these two immune cells, which was consistent with the results from GEPIA. In the GEO database, the data indicated that there was a significant difference in immune cell infiltration between MESO and normal tissues, which made our results more reliable. The concept of immunophenotypic heterogeneity of MESO is worthy of further exploration to find new treatment strategies to improve the clinical outcomes of patients with this disease.

Our study has several limitations that should be acknowledged. Firstly, our study only analyzed the infiltration of immune cells between normal and MESO tissues, without paying attention to the differences between subtypes. Thus, immune cell infiltration based on histological subtypes of MESO needs further exploration. Secondly, our conclusions were based on bioinformatics methods. Therefore, further molecular biological experiments should be presented to confirm the biological functions of COL1A1 in MESO. Last but not least, in public databases, datasets on MESO are relatively scarce. More relevant clinical samples should be collected for MESO research. The research needs to be verified in a larger cohort of MESO.

## 5. Conclusion

With the help of bioinformatics analysis, we found that there was a distinct difference in the infiltration of immune cells between MESO and normal tissues. The differences in immune infiltration were significantly associated with MESO patients' prognosis. A PPI network was constructed for MESO based on DEGs. A hub gene, COL1A1, was identified, which was highly expressed in MESO tissues than normal tissues. COL1A1 expression was in association with the immune cell infiltration across MESO samples. Patients with high COL1A1 expression usually indicated a poorer prognosis than those with its low expression. Thus, COL1A1 could become a potential immune-related prognostic marker for MESO, which requires to be validated in a larger MESO cohort.

## Figures and Tables

**Table 1:** The ranking of hub genes according to different methods using cytoHubba.

MNC	Degree	EPC	Bottleneck	Closeness	Radiality	Betweenness	Stress
COL1A1	COL1A1	SPP1	SPP1	SPP1	SPP1	PPARG	SPP1
MCM4	PPARG	COL1A1	PPARG	COL1A1	PPARG	SPP1	COL1A1
MCM2	MCM4	RUNX2	CDH2	PPARG	COL1A1	COL1A1	PPARG
BUB1	MCM2	MCM4	COL1A1	RUNX2	RUNX2	PECAM1	CDH2
ASPM	SPP1	COL3A1	RUNX2	PECAM1	PECAM1	RUNX2	RUNX2

Abbreviation: MCC: maximal clique centrality; MNC: maximum neighborhood component; Degree: node connects degree; EPC: edge percolated component.

**Table 2:** Cox proportional hazard model of MESO among 84 patients.

Parameter	Coef	95%CI_lower	95%CI_upper	HR	*p* value
Age·	0.029	0.998	1.062	1.029	0.07
Gender (male)	-0.25	0.415	1.464	0.779	0.438
Race (Black)	0.705	0.101	40.397	2.024	0.644
Race (White)	-0.116	0.114	6.935	0.89	0.912
Stage I	-0.151	0.345	2.14	0.86	0.745
Stage II	-0.142	0.385	1.954	0.868	0.732
Stage III	-0.483	0.24	1.588	0.617	0.317
Purity	-0.297	0.248	2.223	0.743	0.595
COL1A1	0.28	1.134	1.544	1.323	<0.0001

Abbreviation: coef: coefficient; CI: confidence interval; HR: hazard ratio.

## Data Availability

The data used to support the findings of this study are included within the supplementary information files.

## Abbreviations

MESO: Mesothelioma
DEGs: Differentially expressed genes
PPI: Protein-protein interaction
GSEA: Gene Set Enrichment Analysis
MPM: Malignant pleural mesothelioma
TILs: Tumor-infiltrating lymphocytes
GEO: Gene expression omnibus
TCGA: The Cancer Genome Atlas
FC: Fold change
CIBERSORT: Cell type identification by estimating relative subsets of RNA transcripts
PPI: Protein-protein interaction.

## Supplementary Materials

Supplementary Materials Supplementary Table 1: the clinical information of MESO patients in the GSE51024 dataset.
