# Supplementary material for: COL1A1 Is a Potential Prognostic Biomarker and Correlated with Immune Infiltration in Mesothelioma
Source: Biomed Res Int. 2021 Jan 4;2021:5320941. doi: 10.1155/2021/5320941 (PMC7803428; doi:10.1155/2021/5320941)
Supplement: Supplementary Materials — Supplementary Table 1: the clinical information of MESO patients in the GSE51024 dataset. [file 5320941.f1.docx]

Supplementary Table 1. The clinical information of MESO patients in the GSE51024 dataset.

| Number of patients | 53 |
| --- | --- |
| Median Age (years) | 72 |
| Gender |  |
| Male | 44 |
| Female | 9 |
| Histology Type |  |
| Epitheloid | 35 |
| Biphasic | 12 |
| Sarcomatoid | 6 |
| Pathological TNM Stage |  |
| Stage I | 2 |
| Stage II | 2 |
| Stage III-IV | 49 |
| Pathological Tumor (T) |  |
| T1 | 2 |
| T2 | 6 |
| T3 | 39 |
| T4 | 6 |
| Pathological Lymph Node (N) |  |
| N0 | 23 |
| N1 | 8 |
| N2 | 19 |
| N3 | 3 |
| Pathological Metastasis (M) |  |
| M0 | 52 |
| M1 | 1 |
| Treatment* |  |
| Neoadjuvant | 11 |
| Adjuvant | 33 |

* Chemotherapy and/or radiotherapy.
